# Conformal CVD-Grown MoS_2_ on Three-Dimensional Woodpile Photonic Crystals for Photonic Bandgap Engineering

**DOI:** 10.1021/acsaom.3c00055

**Published:** 2023-05-10

**Authors:** Mike P. C. Taverne, Xu Zheng, Yu-Shao Jacky Chen, Katrina A. Morgan, Lifeng Chen, Nadira Meethale Palakkool, Daniel Rezaie, Habib Awachi, John G. Rarity, Daniel W. Hewak, Chung-Che Huang, Ying-Lung Daniel Ho

**Affiliations:** † Department of Mathematics, Physics & Electrical Engineering, Northumbria University, NE1 8ST Newcastle upon Tyne, U.K.; ‡ Department of Electrical and Electronic Engineering, University of Bristol, BS8 1UB Bristol, U.K.; ¶ Optoelectronics Research Centre, University of Southampton, SO17 1BJ Southampton, U.K.

**Keywords:** direct laser writing, two-photon lithography, chemical vapor deposition, chalcogenide materials, photonic bandgap, three-dimensional photonic crystals

## Abstract

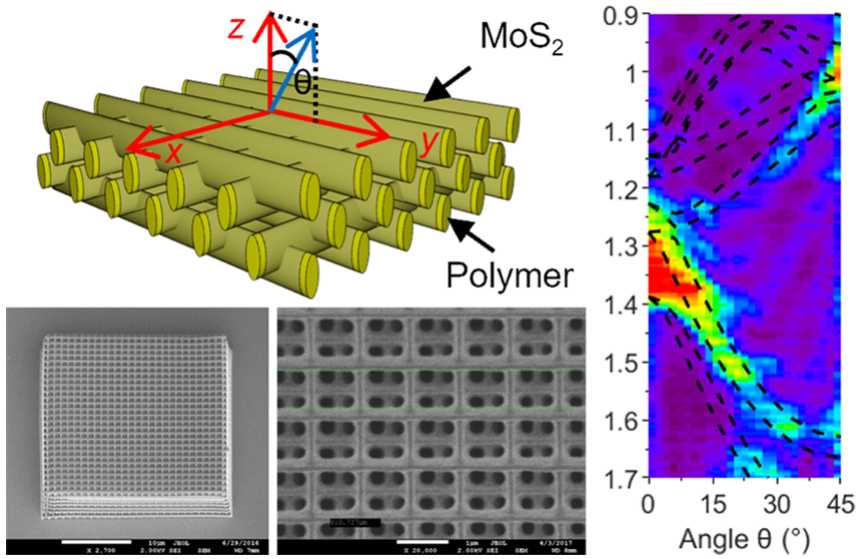

To achieve the modification of photonic band structures and realize the dispersion control toward functional photonic devices, composites of photonic crystal templates with high-refractive-index material are fabricated. A two-step process is used: 3D polymeric woodpile templates are fabricated by a direct laser writing method followed by chemical vapor deposition of MoS_2_. We observed red-shifts of partial bandgaps at the near-infrared region when the thickness of deposited MoS_2_ films increases. A ∼10 nm red-shift of fundamental and high-order bandgap is measured after each 1 nm MoS_2_ thin film deposition and confirmed by simulations and optical measurements using an angle-resolved Fourier imaging spectroscopy system.

## 1 Introduction

3D photonic crystals have a large range of applications due to their potential for showing omnidirectional photonic bandgaps.^1^ This enables the creation of low-loss waveguides,^2,3^ light bending,^2,4^ waveguide splitters,^3^ optical diodes,^5^ negative refraction,^6^ and self-collimation^7^ for example. Another possibility is the confinement of light in a small volume,^8−10^ which has several applications in quantum technologies such as single-photon sources^11,12^ and spin photon interfaces.^13^

Such 3D photonic crystals can be fabricated using direct laser writing (DLW).^14^ However, this technique is limited to the fabrication of low-refractive-index polymeric materials, which leads to partial narrow bandgaps. Additional postprocessing, such as single^15,16^ or double inverse backfilling^17^ with high-refractive-index materials, is therefore usually necessary to obtain a full bandgap. Aside from full bandgaps, high-refractive-index contrast can also be used to tune photonic bands, open and enlarge bandgaps, and control the dispersion of photonic crystals. This can be done using conformal coating with high-refractive-index materials. Such efforts have previously been made in one-dimensional (1D) photonic crystals^18^ and butterfly wings (naturally occurring photonic crystals).^19^ In 2005, Biswas et al. successfully demonstrated a full photonic bandgap at 6–7 μm by coating a polymeric three-dimensional (3D) woodpile structure^20,21^ with titania (TiO_2_).^22^ Furthermore, in 2010, Buso et al.^23^ demonstrated bandgap shifts from a polymeric woodpile photonic crystal after coating it with successive 20 nm CdS layers, with a shift of 375 nm after an 80 nm CdS coating.

Herein, a similar process is applied but at a smaller scale (photonic bandgap at ∼1.3 μm) and using thin (<10 nm) molybdenum disulfide (MoS_2_) films. MoS_2_ has been gaining attention as an emerging material in various applications due to its properties such as a semiconducting nature, high on/off current ratio (10^8^) at room temperature, and mobility of about 200 cm^2^ (V s)^−1^.^24−27^ Bulk MoS_2_ can act as an indirect bandgap semiconductor with a high refractive index comparable to that of silicon, while monolayer MoS_2_ possesses a direct bandgap, which makes it a good candidate for a light emitting medium. In this paper, we treat this material as bulk and focus on its high-refractive-index property. Conformally coated polymeric templates of photonic crystal structures could be used to enhance nonlinear optical responses of materials, such as MoS_2_,^28^ for highly efficient 3D all-optical switching for fast and energy-efficient memory devices.^29,30^ MoS_2_ is also an infrared transparent material and could therefore be a good candidate for mid-infrared sensing applications.^31,32^

For the polymeric templates, a woodpile structure^33^ was chosen, as it is a well-known simple photonic crystal that can be easily fabricated by using layer by layer lithographic approaches^34−36^ (enabling mass production) or DLW.^37^ Alternatively, rod-connected diamond structures, which have larger bandgaps for identical index contrasts,^38^ could be used instead in future work. However, these can only be made by DLW and so are limited to a slow writing process.

**Figure 1:**
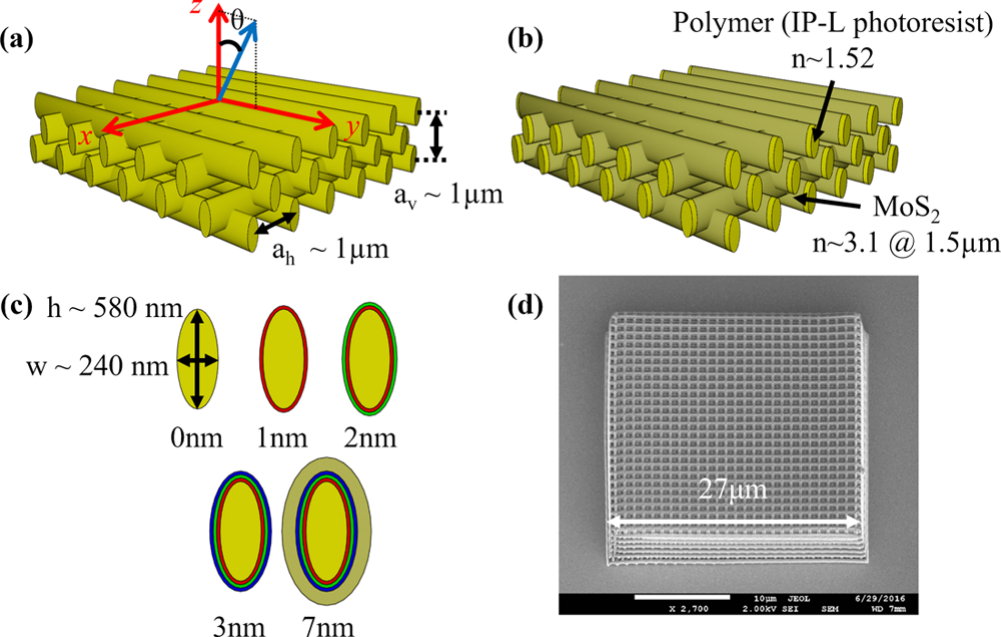
Simplified schematics of: (a) a noncoated woodpile template, (b) a thin film MoS_2_-coated woodpile template, and (c) the resulting rod cross sections after each MoS_2_ thin film coating. Different colors are used to indicate the thin films coated in sequence. The scale of the thin film coatings relative to the inner polymer ellipse has been increased for clarity. (d) SEM image of the fabricated woodpile template.

The fabrication procedure involves steps wherein 3D polymeric woodpile templates (see Figure 1) are fabricated by DLW, followed by low-temperature chemical vapor deposition (CVD) of MoS_2_ on them.^39^ The optical properties of the composite structures are then examined by measuring reflection spectra changes after successive thin film coatings via our in-house-built angle-resolved Fourier imaging spectroscopy (FIS) system.^15,40,41^

## 2 Geometry Description

Figure 1(a–c) illustrates the design of the woodpile template and the thin film coatings, and Figure 1(d) shows a scanning electron microscope (SEM) image of the fabricated woodpile template. The parameters of the fabricated body-centered cubic (BCC) woodpile^42−44^ are given as *a*_*v*_ = *a*_*h*_ = 1 μm, where *a*_*v*_ is the vertical period and *a*_*h*_ is the lateral rod distance. The rod height *h* ≈ 580 nm is larger than the rod width *w* ≈ 240 nm, due to the aspect ratio limitations in the write process.^45^ The number of horizontal layers was *N*_*layers*_ = 24, while the number of rods in each layer alternated between *N*_*rods*_ = 27 and *N*_*rods*_ = 28, with the top two layers having 27 rods each. These parameters correspond to a BCC woodpile with 27 × 27 × 6 periods. The size of the woodpile was 27 × 27 μm.

## 3 Fabrication Methods

**Figure 2:**
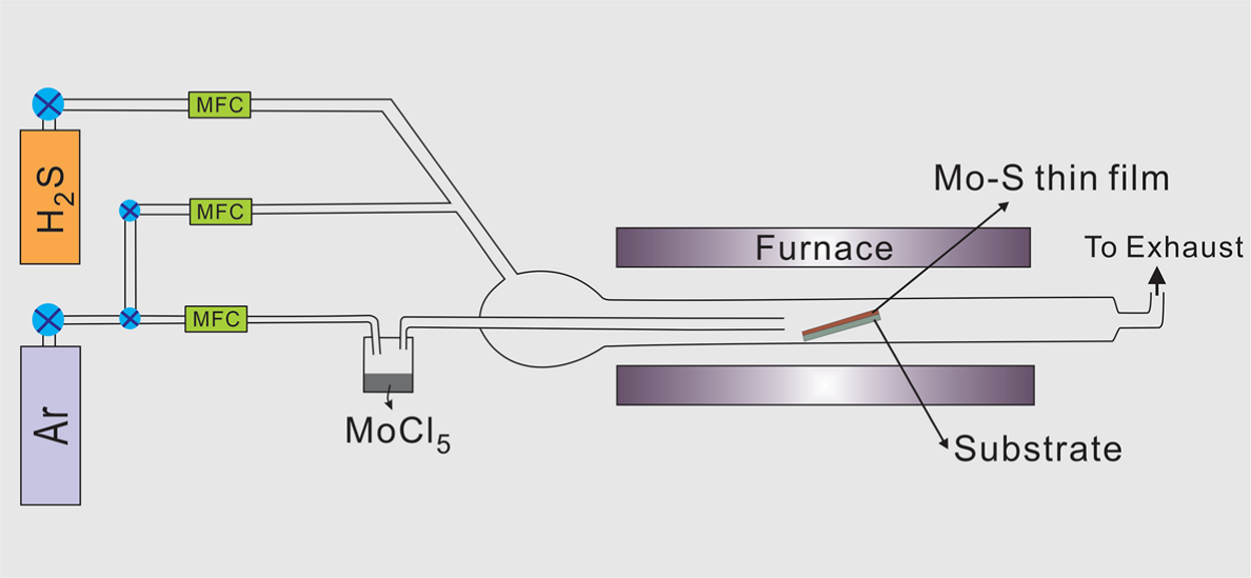
Schematic diagram of the chemical vapor deposition setup used for MoS_2_ thin film deposition.

The 3D polymeric woodpile templates (see Figure 1) were first fabricated by two-photon polymerization DLW (see Methods section), using a negative photoresist (IP-L 780, Nanoscribe GmbH). They were then subsequently coated with molybdenum disulfide (MoS_2_) thin films using chemical vapor deposition (CVD), to achieve high-refractive-index contrast composites. The schematic diagram of the chemical vapor deposition setup used for MoS_2_ thin film deposition in this project is shown in Figure 2 (modified from reference (39)).

First, a thin film of Mo–S is deposited on the woodpile templates’ substrates using MoCl_5_ as precursor, kept in a bubbler, and delivered by Ar gas through a mass flow controller (MFC) to react with a H_2_S/Ar gas mixture through another two MFCs at room temperature. The as-deposited Mo–S thin film could contain excess sulfur (S) and a small portion of unreacted Cl atoms. Hence, second, in order to convert the Mo–S–Cl composition to pure MoS_2_, an annealing step in the H_2_S atmosphere is needed. This annealing step is normally done at high temperature for several hours with the following gases: H_2_S/Ar, 6% H_2_/Ar. This process can be described with the following chemical formula:
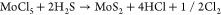


The deposition time, annealing temperature, and the annealing time are the parameters affecting the thickness and the refractive index of the resulting MoS_2_ thin film. Here, a thin film deposition for 30 min at room temperature and a following annealing treatment at 250 °C for 3 h are chosen to achieve a ∼1 nm MoS_2_ deposition while avoiding any obvious thermal deformation of the woodpile templates. The first three layers of coatings were done for 30 min each, while the last coating was done for 120 min in order to observe a bigger shift, i.e., total deposition times of 30, 60, 90, and 210 min for the four coatings, leading to the following expected coating thicknesses: 1, 2, 3, and 7 nm.

The refractive index was measured using ellipsometry for a 10 nm thin film. For a wavelength of 1500 nm, the measured refractive index is *n* ≈ 3.1, the extinction coefficient is *k* ≈ 0.62 (Figure S1), and the absorption is around 7% (Figure S2). Hence, for the simulations, the refractive index for the MoS_2_ coatings was taken as *n* = 3.1. The material composition of the final MoS_2_ layer coated on the surface of the woodpile was estimated using energy density X-ray spectroscopy (EDX) (Figure S3). The atomic percentage of sulfur and molybdenum was found to be in the ratio of approximately 2.5, confirming the presence of a mixture of MoS_2_ (crystalline) and MoS_3_ (amorphous).

The coating film thickness was estimated to be around 35 nm on the substrate by taking a transmission electron microscopy (TEM) image of a cross section slice and performing EDX mapping on it (Figures S4 and S5). However, the film thickness on the woodpile is expected to be less due to the increased surface area. Based on the woodpile parameters, the total surface area of the woodpile was calculated to be *A*_*woodpile*_ ≃ 17 333 μm^2^, while the substrate area it sits on is *A*_*substrate*_ = (27 μm)^2^ = 729 μm^2^. This corresponds to a ratio *r* = *A*_*woodpile*_/*A*_*substrate*_ ≃ 24. Assuming the deposited volume per substrate area is the same and the coating is homogeneous, we would therefore expect a coating thickness of 35 nm/*r* ≃ 1 nm. Given the measurement results, the coating thickness on the woodpile is therefore likely to be between 1 and 35 nm.

## 4 Results and Discussion

**Figure 3:**
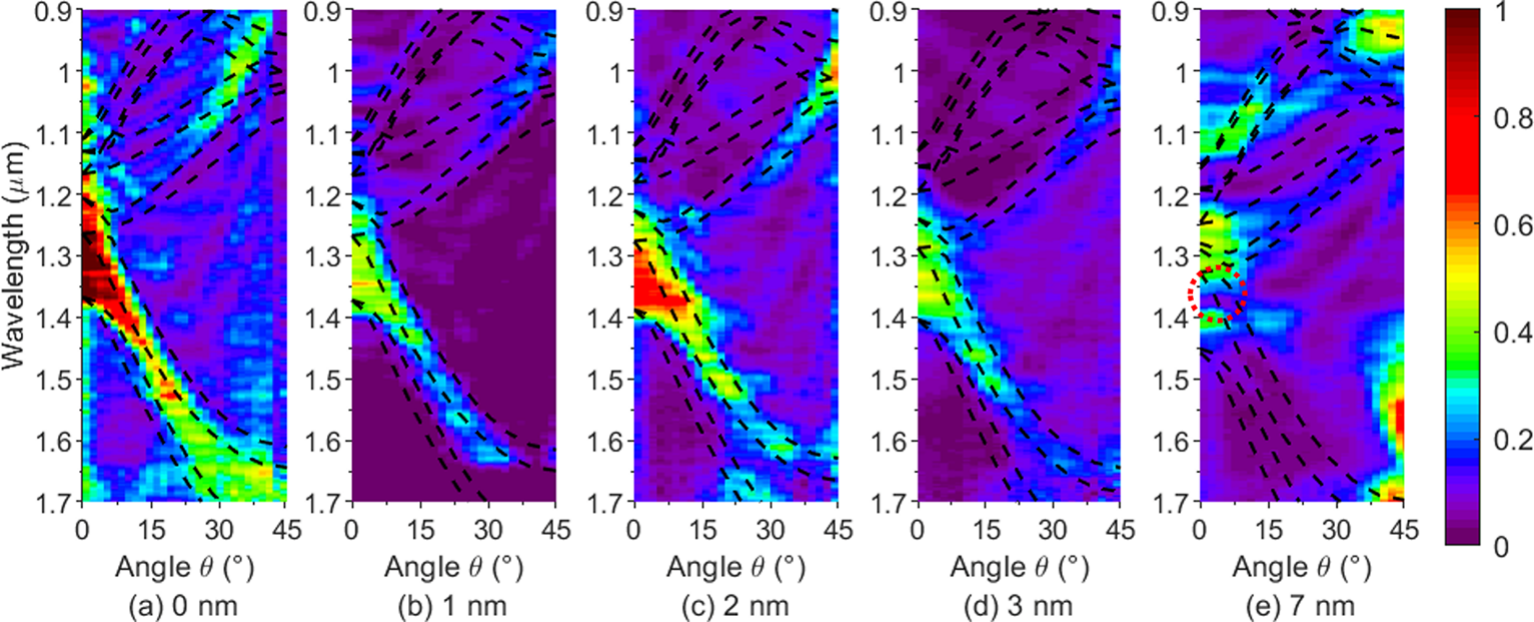
Measured angle-resolved reflection spectra of woodpile structures: (a) noncoated and (b) 1 nm, (c) 2 nm, (d) 3 nm, (e) 7 nm MoS_2_ thin-film-coated. Dashed lines indicate their corresponding photonic band structures calculated via the PWE method. The red dashed circle in (e) highlights the splitting of the fundamental bandgap.

The resulting structures were analyzed with an FIS system,^15^ where reflection intensity is collected as a function of angle and wavelength (see Methods section), using white light illumination across the 0.9–1.7 μm band. Figure 3 shows the results for S-polarized light (electric field along the X direction (Figure 1a)) measured as a function of the angle θ with increasing coating thicknesses from left to right. Dashed lines indicate their corresponding photonic band structures calculated via the plane-wave expansion (PWE) method using the MIT Photonics Bands software,^46^ with refractive index values of *n*_*IP*–*L*_ = 1.52 for the polymerized IP-L^41^ and  for the MoS_2_ coatings. As can be seen, both the fundamental and the high-order gaps exhibited red-shifts after each deposition.

**Figure 4:**
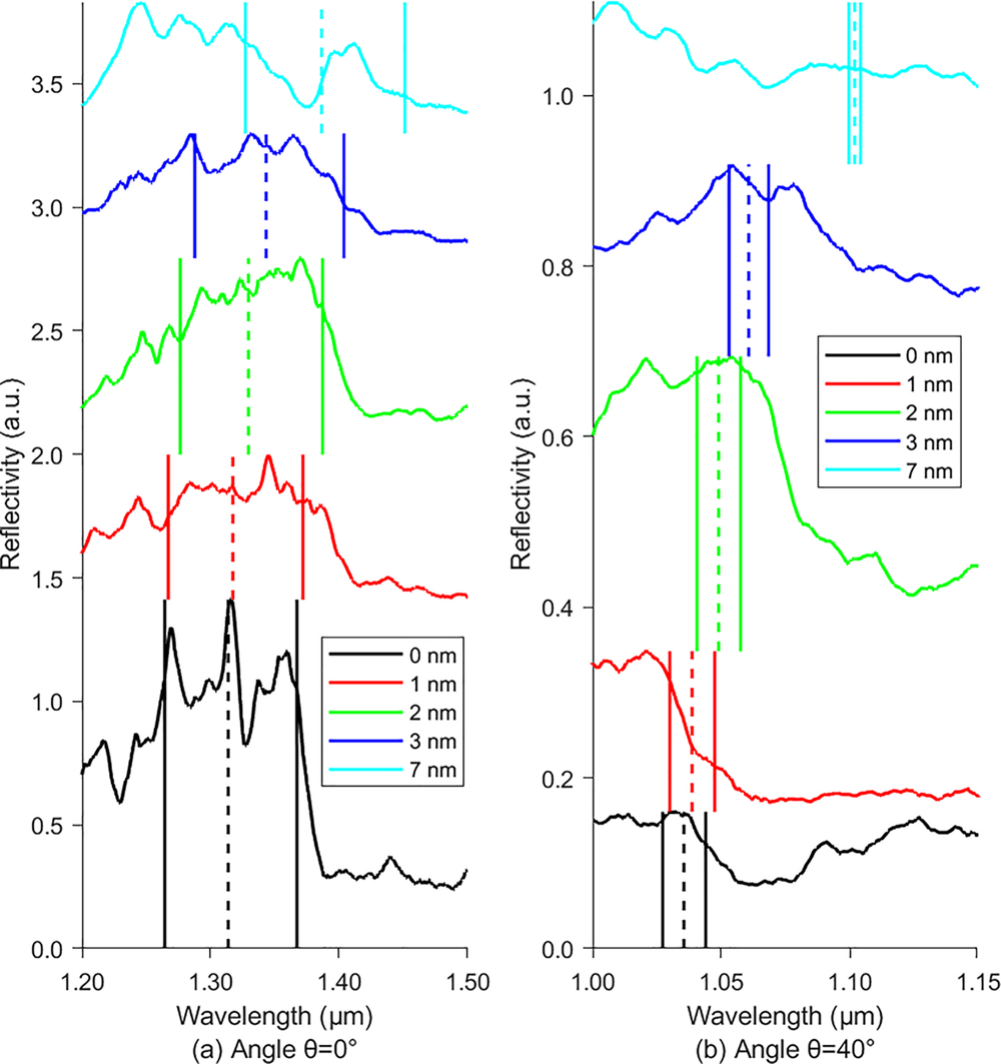
Measured reflection spectra from the woodpile template with varying MoS_2_ thin film coating thicknesses: 0 nm (noncoated, solid-black), 1 nm (solid-red), 2 nm (solid-green), 3 nm (solid-blue), and 7 nm (solid-cyan) for angles of (a) 0° and (b) 40°. The vertical lines indicate the bandgap centers (dashed lines) and edges (solid lines) calculated using PWE of the gaps between bands 2 and 3 at 0° in (a) and between bands 6 and 7 at 40° in (b) for the corresponding coating thicknesses: 0 nm (noncoated, black), 1 nm (red), 2 nm (green), 3 nm (blue), and 7 nm (cyan).

Direct comparisons of the reflection spectra at normal incidence (for the fundamental bandgap, between bands 2 and 3) and at 40° incidence (for the higher order bandgap, between bands 6 and 7) are shown in Figure 4. The vertical dashed and solid lines indicate the corresponding bandgap centers and edges calculated using PWE. We show the results at 40°, because this is the largest angle measurable with our existing setup. At 45°, there are technical limitations, due to being at the edge of the objective lens. As can be seen in Figure 3, there are strong reflection anomalies at large angles, especially in Figure 3e. These are due to glancing angle reflections, which are difficult to remove in our background correction methods.

As shown in Figure 3, each measurement exhibited two main bands: first, a band red-shifting with increasing angle, corresponding to a grating-like behavior, and second, a band blue-shifting with increasing angle, corresponding to a behavior similar to that of a distributed Bragg reflector (DBR). As the coating thickness increases, both main bands red-shift as a result of the increasing effective refractive index. In the case of P-polarized incident light, the bandgaps are generally thinner, due to the weaker reflection of P-polarized light at non-normal incidence angles.^37^ In addition, the grating-like red-shifting band fades in favor of the DBR-like blue-shifting band (see Figure S6).^42^ Randomness in coating thickness is likely the cause of reduced band visibility in samples with the thickest coatings. At low thickness, it is clear that there is a good match between bands and measurements. Deviations will be due to fabrication errors and particularly the surface roughness, which increases with increasing coating thickness, thereby reducing the contrast between band and background. Furthermore, there is almost certainly a variation in coating thickness with depth into the sample, which effectively washes out the detail, particularly at high angle. The variability is evident, particularly at higher coating thickness.

From the sample with a 7 nm coating thickness, a splitting of the fundamental bandgap was observed (red dashed circle in Figure 3e). Moreover, it was not exhibiting the fundamental grating-like band. Rather than a shift in the bandgap, a change in the band structure was observed with a band at ∼1.4 μm splitting off from the main bandgap moving toward the red. Although these trends are clearly visible in the 2D plots (Figure 3), it is a lot harder to discriminate the extent of red-shift from single angle measurements due to the complex substructure of the band.

Figure 4a represents the measured reflection spectra corresponding to the reflection at 0°. When considering the long wavelength band edges (right vertical solid lines), a 5 nm red-shift of the fundamental bandgap (∼1.3 μm) was observed at normal incidence after the first 1 nm MoS_2_ coating, followed by another two red-shifts of 15 and 17 nm after the second and the third depositions, respectively. At 0°, the long wavelength band edges of the measured reflection peaks match the calculated bandgap edges (right side). However, as the thickness increases to 7 nm, the splitting of the band puts the red-shifted peak close to the predicted red-shifted band. In the thinner coated structures, it is difficult to see the substructure of the band, making it difficult to confirm the small shifts predicted. In the reflection measurement corresponding to the 40° angle (Figure 4b), narrower peaks and distinct red-shifts at the long wavelength band edges can be seen. However, it was difficult to accurately measure the exact band centers and edges. For the 1 nm coating (red curve), there is a first strongly visible band edge around 1.03 μm, followed by a smaller drop around 1.05 μm, corresponding to the expected band edge. At 7 nm coating thickness, the expected reflection peak is not visible. This is due to the corresponding bandgap being much narrower than the ones at thinner coatings as well as a high-angle anomalous scattering peak that suppresses it.

**Figure 5:**
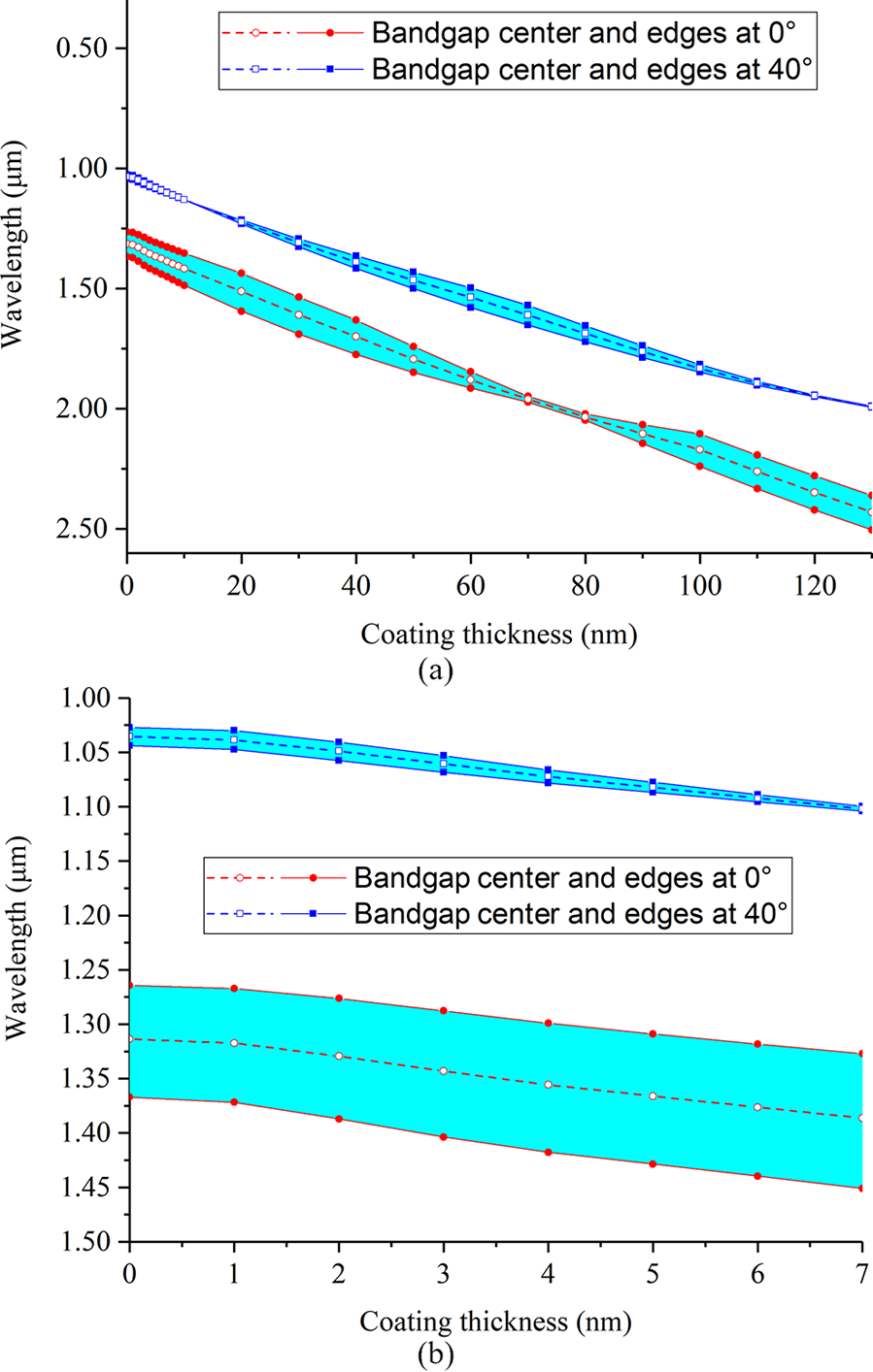
Calculated bandgap centers and edges vs coating thickness for the fundamental bandgap (bands 2–3) at normal incidence and the higher order bandgap from band 6 to 7 at an incident angle of 40° via the PWE method. The solid lines indicate the band edges, while the dashed lines indicate the corresponding center (in terms of frequency). Thickness from (a) 0 to 130 nm and from (b) 0 to 7 nm.

To predict the bandgap shifts for larger coating thicknesses, multiple PWE simulations were run, and the midgap position and band edges of the fundamental bandgap (bands 2–3) at normal incidence and the higher order bandgap from band 6 to 7 at 40° incidence were calculated for each simulation. Figure 5 shows the corresponding results. This confirms the observed red-shifts with increasing coating thickness. The bandgap positions shift almost linearly with coating thickness, varying from 1.3 to 2.4 μm for the fundamental bandgap at normal incidence and from 1 to 2 μm for the higher order bandgap at 40° incidence as the coating increases from 0 to 130 nm.

## 5 Conclusion

Polymeric woodpile templates have been successfully fabricated and coated with multiple successive layers of MoS_2_. Simulations of the structures show partial bandgaps in the short wave infrared region with a 10 nm red-shift occurring after each addition of 1 nm of MoS_2_ thin film deposition. Measurement results are less easy to interpret. The 2D plots obtained from measuring scattering spectra as a function of angle (wave vector k⃗) show band structures with a small red-shift observed with increasing thickness of the coating. By matching these results to the band structure simulations, this becomes more apparent and helps calibrate the actual film thickness. However, the substructure arising from the convergence of several bands makes it difficult to measure the red-shifts in the zero degree spectral plot. As thickness of the coating increases, the bands become less distinct, which we ascribe to variations in coating thickness in the structure, which smear the bandstructure. However, a clear red-shifted band was observed in the thickest sample. In future work, we aim to increase coating thickness to show clearer red-shifts and strong wide angle bandgaps. The potential of our approach opens the way for developing a process to reliably engineer photonic bandgap materials through thin film deposition of MoS_2_. Such conformally coated polymeric templates of photonic crystal structures could be used to enhance nonlinear optical responses for optical switching and sensing applications. Additionally, this work represents a step forward in determining the thicknesses of 3D wavelength scale structures with nanocoating thin films by using an angle-resolved FIS system and expands the library of coating nanomaterials, leading to a wide range of future quantum and nanophotonic applications.

## A Methods

### A.1 Two-Photon Polymerization (2PP)

The Nanoscribe machine (*Nanoscribe Photonic Professional*) is a DLW system based on the 2PP method for the fabrication of arbitrary 3D nanostructures in photoresists such as IP-L.^47−49^ The laser beam is produced by a femtosecond fiber laser (center wavelength: 780 nm, average output power: 155 mW, peak power: 25 kW, pulse duration: 94 fs, repetition rate: 80 MHz) and focused into the photoresist through an oil-immersion objective lens with a NA of 1.4 and 100× magnification. The photoresist is drop-cast onto a substrate glass, which is glued on the piezoelectric 3D scanning stage.

### A.2 Fourier Imaging Spectroscopy (FIS)

An identical system as that of our previous work^15,40^ has been used here. This home-built Fourier imaging spectroscope uses a 4× objective lens to collimate a fiber (200 μm diameter) coupled white light source (Bentham Ltd. WLS100 300–2500 nm), focusing the light beam with an NA = 0.9, 60× objective lens on the sample. The detection plane is a projection image for the backfocal plane of the objective lens. This image is scanned by a fiber (105 μm diameter) attached to an *x*-*y* motorized stage, and the other end of the fiber is connected to a spectrometer (Ocean optics NIRQuest512), which has a 900–1700 nm spectrum range. The angular resolution of the system is ∼2° per scan step.

## Supporting Information Available

Additional information about the optical properties of MoS_2_; EDX and TEM analysis of the thin film coating; reflection spectra for S and P-polarized incident light (PDF)
