# Supplementary material for: Conformal CVD-Grown MoS2 on Three-Dimensional Woodpile Photonic Crystals for Photonic Bandgap Engineering
Source: ACS Appl Opt Mater. 2023 May 10;1(5):990–6. doi: 10.1021/acsaom.3c00055 (PMC10226160; doi:10.1021/acsaom.3c00055)
Supplement: Supplementary file 1 — ot3c00055_si_001.pdf [file ot3c00055_si_001.pdf]

Supporting Information: Conformal CVD-grown MoS2 on Three-dimensional Woodpile Photonic Crystals for Photonic Bandgap Engineering

Mike P. C. Taverne,[†,‡] Xu Zheng,[‡] Yu-Shao Jacky Chen,[‡] Katrina A. Morgan,[¶] Lifeng Chen,[‡] Nadira Meethale Palakkool,[†] Daniel Rezaie,[†] Habib Awachi,[†] John G. Rarity,[*,‡] Daniel W. Hewak,[¶] Chung-Che Huang,[*,¶] and Ying -Lung Daniel Ho[*,†,‡]

†*Department of Mathematics, Physics & Electrical Engineering, Northumbria University, NE1 8ST, Newcastle upon Tyne, UK*

‡*Department of Electrical and Electronic Engineering, University of Bristol, BS8 1UB, Bristol, UK*

¶*Optoelectronics Research Centre, University of Southampton, SO17 1BJ, Southampton, UK*

E-mail: john.rarity@bristol.ac.uk; cch@soton.ac.uk; daniel.ho@northumbria.ac.uk

1 Coating film characterization

1.1 Optical properties

The refractive index and extinction coefficient of the MoS_2 films are examined via ellipsometry using a 10 nm MoS_2 thin film on a 300 nm silica (SiO_2) coated silicon (Si) wafer substrate. The results are shown in Figure S1. At 1500 nm, the measured refractive index is $n \sim 3.1$ and the extinction coefficient $k \sim 0.62$.

Figure S2 shows measured transmission, reflection and calculated absorption for a 10 nm MoS_2 thin film deposited on a 170 µm silica coverslip (the same one used in the DLW system for fabricating woodpile templates). The absorption A is calculated as A=1-T-R, where T and R are the normalized transmission and reflection respectively. About 7% absorption can be observed across the wavelengths of interest from 0.9 µm to 1.7 µm.

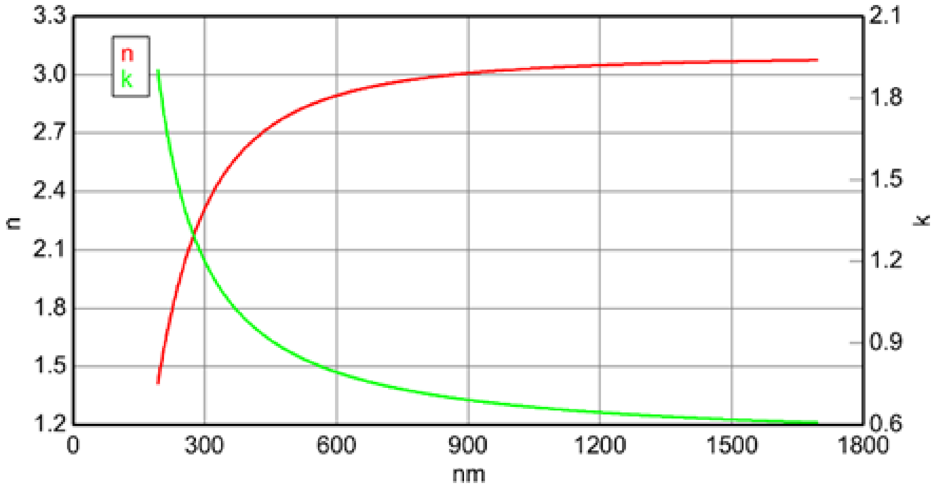

Figure S1: Refractive index and extinction coefficient vs. wavelength measured via ellipsometry for a 10 nm MoS_2 thin film.

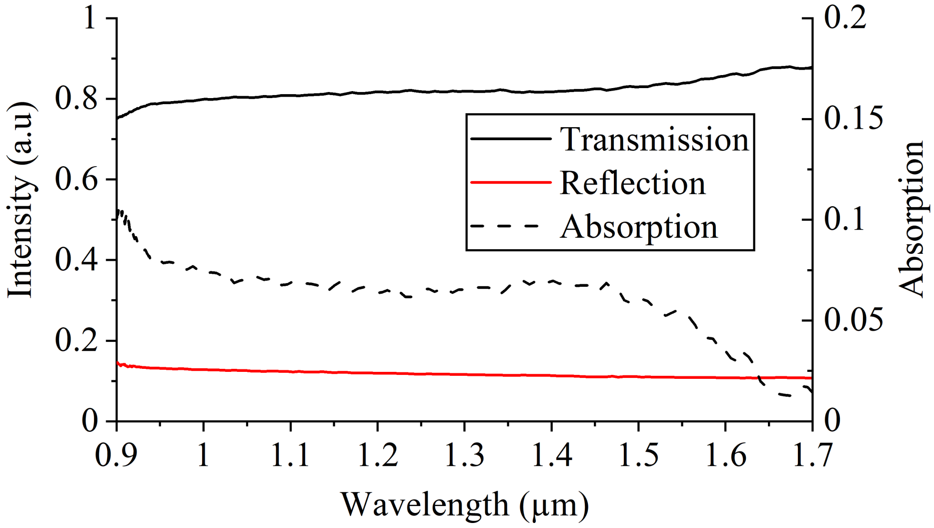

Figure S2: Measured transmission (solid black line, left Y axis), reflection (solid red line, left Y axis) and calculated absorption (dashed black line, right Y axis) of a 10 nm MoS_2 thin film on substrate (170 µm silica coverslip).

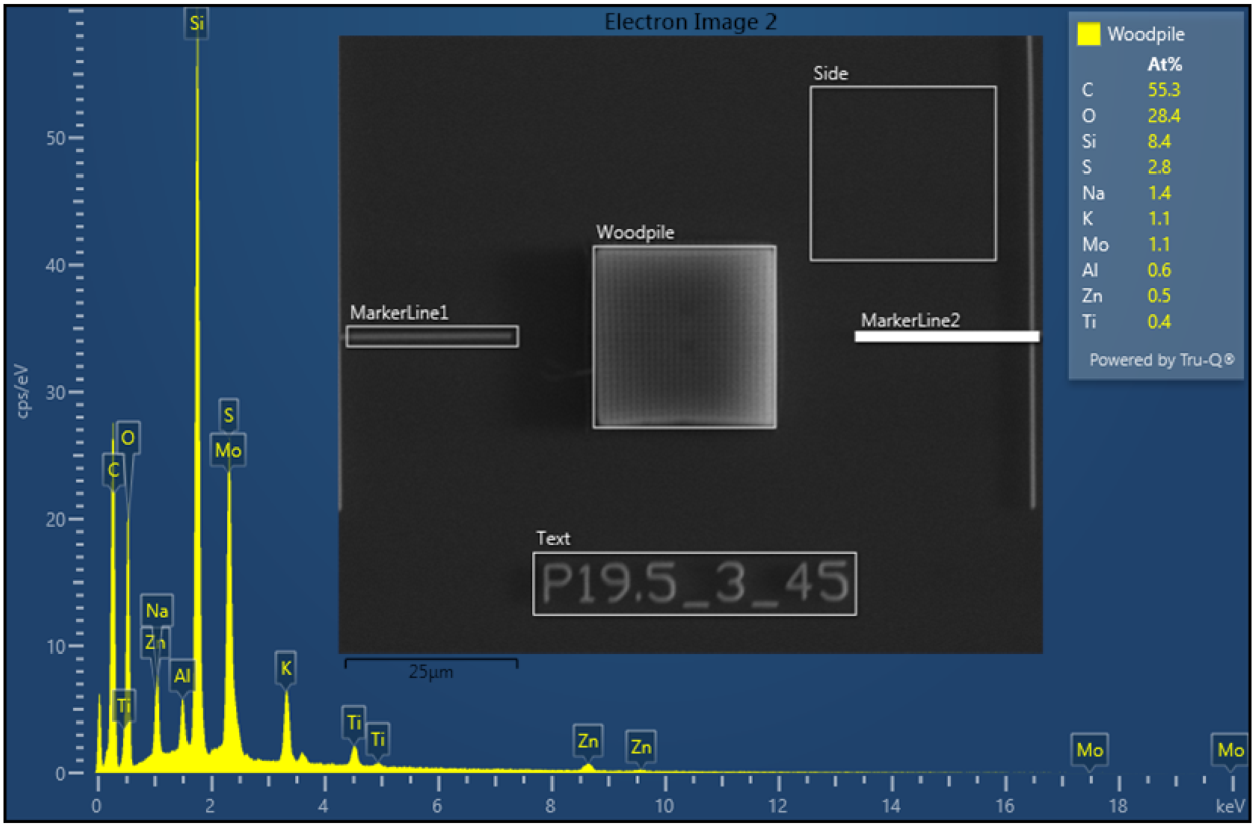

Figure S3: EDX spectrum of the surface of the woodpile after the final MoS_2 coating. The inset shows an SEM image of the sample after the final coating, as well as the scanned area (location "Woodpile").

1.2 Composition

1.3 Thickness on the substrate

In order to estimate the thickness of the coating, a thin vertical cross-section slice was taken near the coated woodpile. Figure S4(a) shows a Transmission electron microscopy (TEM) image of the slice. X-ray spectroscopy (EDX) analysis was then used to identify the elements in it and create the elemental maps for Pt, Ga, Mo ($K\alpha$ and $L\alpha$ transitions), S, Si and O shown in Figures S4(b-h).

Due to the diffusion of the MoS_2 coating into the Pt used to hold the TEM slice, it is difficult to determine the thickness from these elemental maps. The data was therefore projected along the layer direction to create the 1D plots of Figure S5.

The counts for the elements Mo and S show a $\sim 35\ nm$ peak located between the Platinum layer (deposited on top of the substrate during the TEM sample preparation) and the SiO_2 substrate. Some Gallium contamination is visible in the Pt layer, due to the use of a Gallium focused ion beam in the TEM sample preparation. Based on this, the MoS_2 film thickness on the substrate is estimated to be $\sim 35\ nm$. However, the coating thickness on the woodpile is likely to be thinner than on the substrate, due to the 3D structure of it.

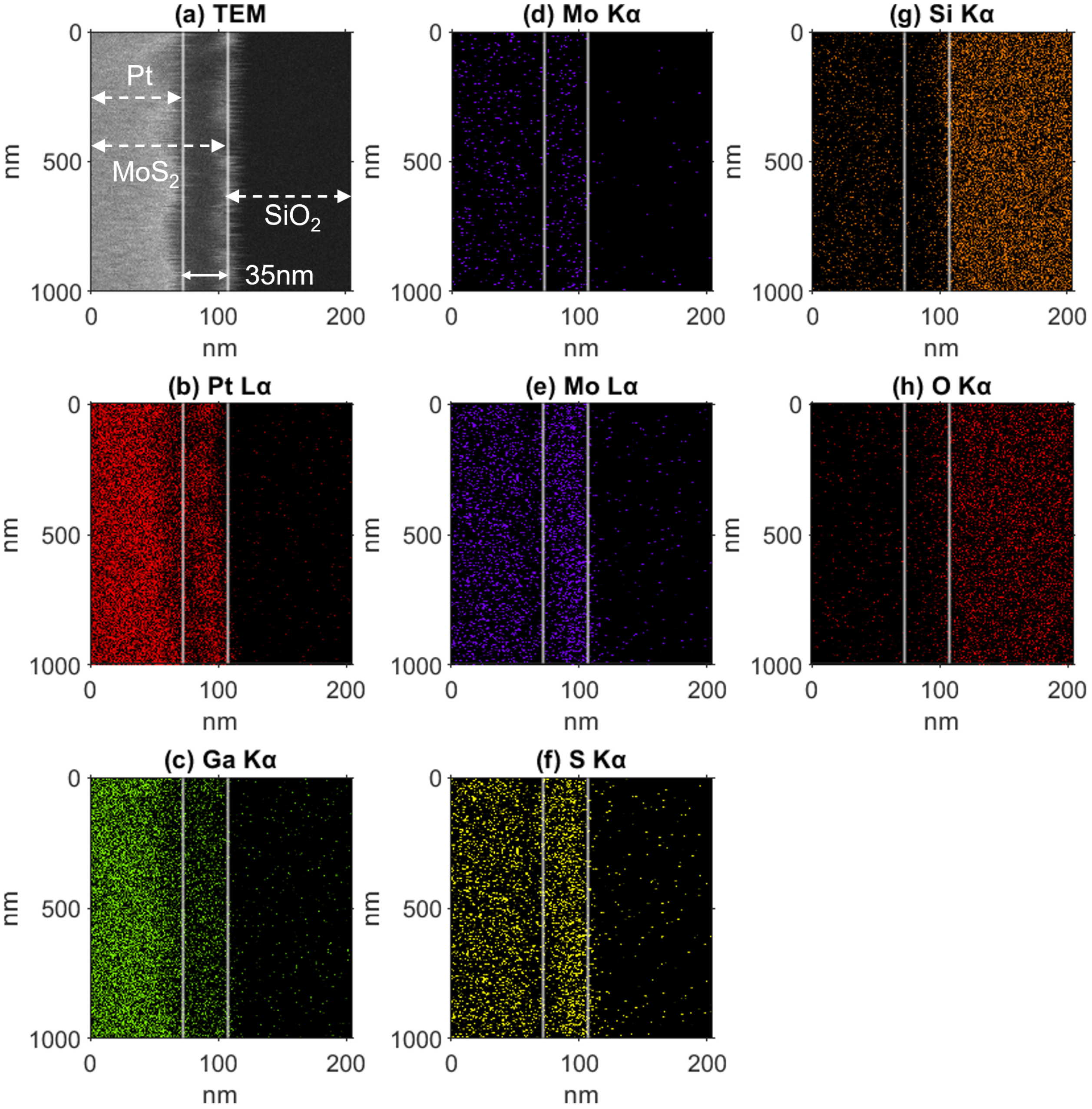

Figure S4: (a) TEM image of a cross-section of the sample surface next to the woodpile. (b-h) Corresponding elemental maps made using EDX analysis for Pt, Ga, Mo ($K\alpha$ and $L\alpha$ transitions), S, Si and O. The vertical white lines indicate a 35 nm wide layer of higher density counts for the elements Mo and S. Note that the X and Y axes do not represent the same length. This was done to maximize available information, while maintaining a small figure size.

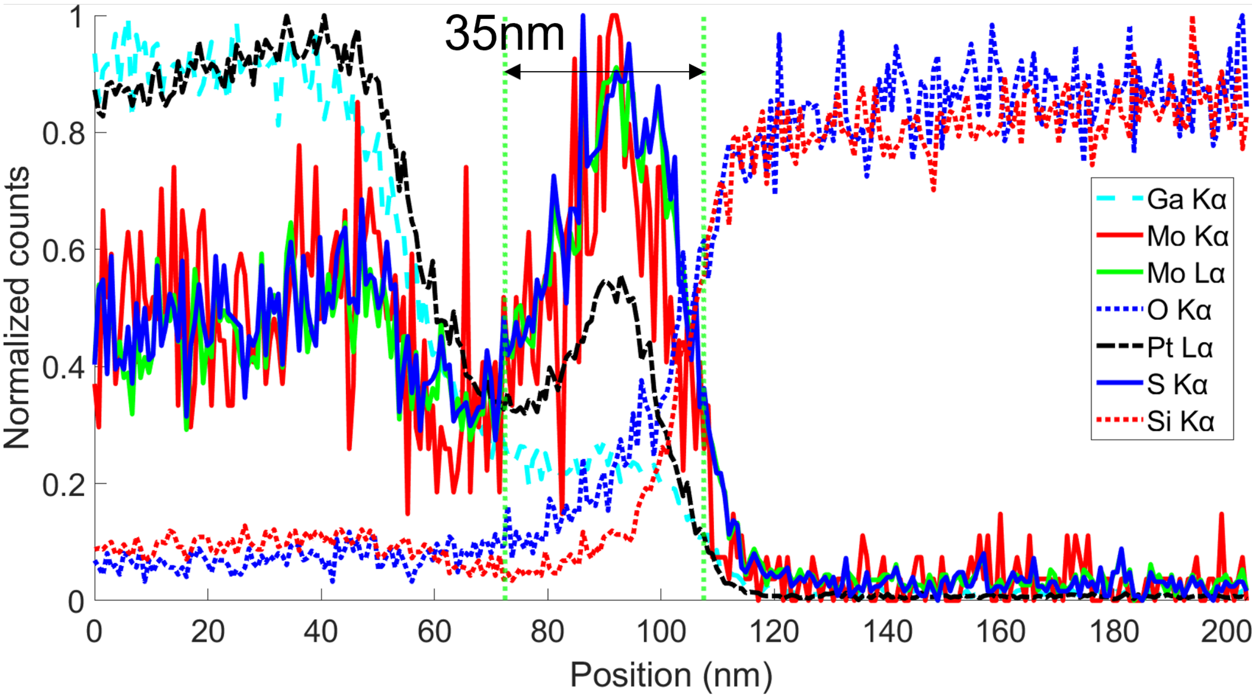

Figure S5: Normalized element counts from the EDX analysis of the cross-section shown in Figure S4(a) as a function of depth. The vertical green dashed lines indicate a 35 nm wide layer of higher density counts for the elements Mo and S.

2 Reflection spectra for S and P-polarized incident light

Figure S6 shows the measurement and simulation results for a BCC woodpile template similar to the one considered in this paper. While the parameters are different, they are similar enough and show a clear difference between the S and P reflectivities of woodpiles in general.

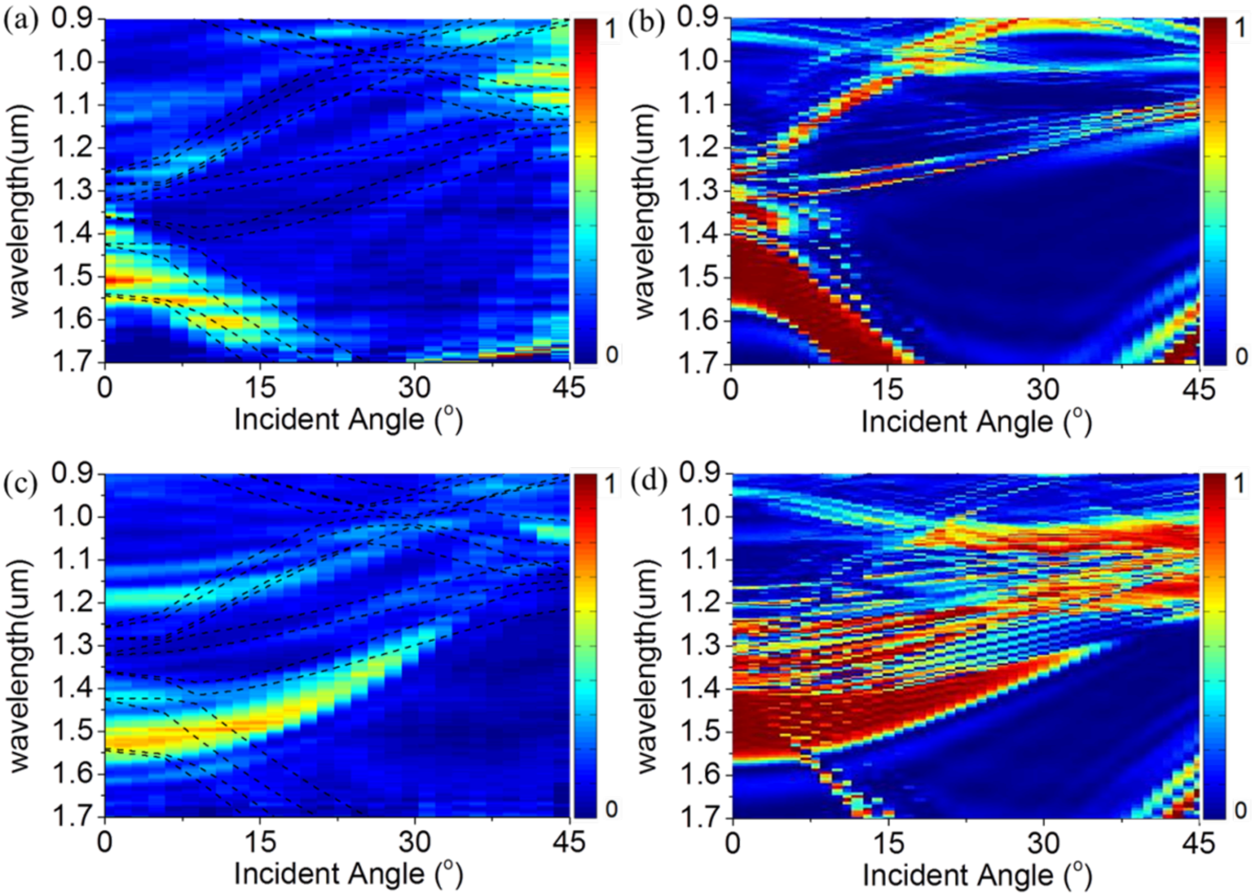

Figure S6: Measured angle-resolved reflection spectra compared with FDTD simulations, for a BCC woodpile template of size 50µm×50µm and with $N'_{layers} = 24$ stacking layers. The vertical period and lateral rod distance in this case are $a'_v = a'_h = 1.14\mu m$. The measured rod height $h' = 640nm$ and rod width $w' = 270nm$. The black dashed lines in (a) and (c) are calculated photonic bands using the PWE method. (a) measured and (b) simulated reflection using S-polarized incident light, (c) measured and (d) simulated reflection using P-polarized incident light.
